# Standard Operating Procedures (SOPs) for Palliative Care in German Comprehensive Cancer Centers - an evaluation of the implementation status

**DOI:** 10.1186/s12904-020-00565-6

**Published:** 2020-05-02

**Authors:** Sarah Lödel, Christoph Ostgathe, Maria Heckel, Karin Oechsle, Susanne Gahr

**Affiliations:** 1 Department of Palliative Medicine, CCC Erlangen – EMN, Universitätsklinikum Erlangen, Friedrich-Alexander-Universität Erlangen-Nürnberg (FAU), Krankenhausstraße 12, 91054 Erlangen, Germany; 2 Palliative Care Unit, Department of Oncology, Hematology and BMT, University Medical Center Hamburg-Eppendorf, Martinistr. 52, 20246 Hamburg, Germany

**Keywords:** Palliative Care, Standard Operating Procedure (SOP), Symptoms, Implementation status, Comprehensive Cancer Center (CCC), Survey

## Abstract

**Background:**

The working group for palliative medicine within the Comprehensive Cancer Center (CCC) network funded by the German Cancer Aid in Germany has developed and published 14 Standard Operating Procedures (SOPs) for palliative care in CCCs. This study analyzed to what extent these SOPs have been implemented in the clinical routine in the CCC network one year after their publication.

**Methods:**

An online-based survey on the implementation status, limitations in daily practice and further themes was conducted between April and July 2018. In total, 125 health professionals in specialized palliative care from all 16 CCC locations were invited to participate. The data were analyzed descriptively using SPSS.

**Results:**

The response rate was 52.8%. More than half of the respondents (57.6%) knew about the free availability of SOPs on the CCC network website. The extent to which each SOP was being used actively in practice by the survey respondents ranged from a low of 22.7% (for the “Fatigue” SOP) to a highest of 48.5% (for the “Palliative Sedation” and “Respiratory Distress” SOPs). The respondents became aware of the SOP through recommendations from colleagues, team meetings or from the head of the department. The SOPs “Respiratory distress of an adult palliative patient” and “Palliative sedation” were perceived as the most practically oriented and understandable. Barriers to use SOPs were mainly limited time resources and lack of knowledge of existence and availability.

**Conclusions:**

In practice, better knowledge about the SOPs and at the same time increased use can be achieved through systematic training or discussion of SOPs in regular team meetings. There is a need to take measures to optimize the implementation in clinical practice.

## Background

In 1994, the German Association for Palliative Medicine was founded and therefore set an important prerequisite for a structured multi-professional and interdisciplinary cooperation. The cornerstone for palliative medicine in Germany had already been laid almost ten years earlier in 1983 in Cologne with the opening of the first palliative ward in Germany. Since then, the discipline has become increasingly important and dynamic. In 2006, the Comprehensive Cancer Center (CCC) network was initiated by the German Cancer Aid. The main objective of this CCC network is to improve cancer research and the treatment of tumor patients, including palliative care. The working group for palliative medicine within the CCC network was established in 2011. An important task was to develop Standard Operating Procedures (SOPs) based on the existing evidence- and consensus-based national S3 guideline for palliative medicine [1]. SOPS are clinically relevant treatment and care algorithms [2] and concrete instruments to support cancer care providers providing palliative care treatments. SOPs in palliative medicine are currently still a rarity in contrast to clinical practice guidelines (CPGs). The term SOP refers to a standardized procedure describing processes step-by-step in text form. The guidelines of the Scientific Medical Societies are systematically developed aids for physicians for decision-making in specific situations. They are based on current scientific knowledge and best practice and provide for more safety in medicine, but should also consider economic aspects. The guidelines are not legally binding for physicians and therefore have neither a liability founding nor a liability releasing effect [3]. While SOPs are standards that are intended to facilitate everyday clinical practice within the clinic, e.g. in the CCCs, guidelines permit general statements and regulations to be made at a higher level.

A survey in 2014/2015 exploring the integration of palliative care in CCCs [4] revealed the lack of palliative care related SOPs in the CCCs. The survey showed that although some SOPs existed, their content as well as their frequency and implementation in clinical practice varied greatly from center to center [2]. This deficiency is demonstrated by the lack of symptom, treatment pathway and process-related recommendations in palliative care. Once that gap was identified, the working group for palliative medicine decided on topics that should be covered by SOPs. The development of the SOPs was based taking into account the national S3 guideline for palliative medicine and current literature. In the palliative medicine working group, topics for SOPs were selected that were most frequently classified as relevant by the palliative care providers surveyed. The representatives from the specific CCCs declared themselves responsible for a topic according to their focus and interest. The developed SOPs were each revised by the authors and then a review was conducted by two representatives from two other CCCs [5]. This resulted in 14 consistent SOPs [6–21] in palliative medicine (see Table 1) were developed within the working group for palliative medicine in the CCC network and published in 2017 [2]. The manuscript reports on a survey conducted to explore the usefulness and adoption of the SOPs published.

**Table 1:** Overview of published SOPs [6–21] status 2018

Topic	
**Symptom-related SOPs**	
Acute state of confusion	
Respiratory distress of an adult palliative patient	
Fatigue	
Pain therapy of palliative patients	
Intestinal passage dysfunctions in palliative medicine	
Inappetence and cachexia	
Nausea and vomiting of palliative patients	
Depression and anxiety in palliative medicine	
**Interventions and processes SOPs**	
Managing and caring for deceased persons	
Treatment of multi-resistant pathogens on the palliative care ward	
Admission criteria for the palliative ward	
Palliative sedation	
Subcutaneous medication and infusions in the adult PC	
**Treatment pathway SOP**	
Treatment and care in the dying phase	

The philosophy was to support staff caring for very ill and dying cancer patients. The SOPs are not specifically addressed to health care professionals working in specialized palliative care, but also to those working in general palliative care in and outside the CCC network. The SOPs have been published as a series in the journal “Der Onkologe” in 2017 [6–20] and are freely available on the website of the CCC network [22]. The extent of the implementation of SOPs with a focus on “symptoms”, “treatment pathways” and “interventions and processes” in the clinical routine of CCCs one year after publication was unclear. This manuscript aims to present the evaluation of the level of implementation of the SOPs in the CCC network considering as well their accessibility and use by medical staff, and the estimation on practicality, comprehensibility and importance by medical staff. The survey was also a measure for quality assurance/improvement in palliative care.

## Methods

Initially, a contact person from each of the 16 CCC within the CCC network sites was asked to give contact details of medical staff working in specialized palliative medicine after prior agreement. In the project no medical research involving patients, family caregivers, or other vulnerable groups was performed. Therefore, according to the federal medical professional code of conduct (§15), approval of the local ethics committee was not required. All participants were informed on the front page of the online-based survey that data would be anonymously stored and proceeded. Participants could only start the online-based survey after agreement to participation on the front page.

This resulted in 125 potential respondents from the various multi-professional teams were included in the online-based survey that took place between May and July 2018. The aim was to obtain an overview of the respondents of the 16 CCCs within the CCC network.

Level of implementation points out which SOPs were used in clinical practice asked in a closed question. In addition respondents were asked to indicate the current level of implementation of palliative care SOPs in their department on a scale with five possible responses. The scale ranged from “already implemented”, “concrete implementation plans”, “discussion of implementation plans” to “no implementation plans yet” and the statement “I don’t know”. Therefore, the level of implementation was asked in two ways, first whether respondents use them or not and after that what current level of implementation status exists. The following items were assessed: mode of becoming aware of the SOP, knowledge of the free availability of the SOPs on the website of the CCC network, storage of and access to the SOP in the specific departments. Frequency of use (not yet, daily, weekly, monthly, quarterly), importance (not important at all, somewhat important, important, very important), practicality (the SOP is practical: yes / no / I do not know the SOP), and comprehensibility (the SOP is understandable: yes / no / I do not know the SOP) were evaluated for each SOP (*n* = 14). Practicality and comprehensibility were asked in a closed question. Furthermore, barriers that hamper the use and possible measures to increase knowledge on and practical implementation of the SOPs were enquired. The data from the survey were analyzed descriptively using IBM SPSS Statistics 21.

## Results

A total of 66 of the 125 persons took part in the survey (52.8%). The respondents were predominantly female (37; 56.1%) and on average 41.9 years old (range: 29 to 62 years).

Of those responding, 45 were physicians, 14 nurses and 7 were from other professional groups. 54 (81.8%) of the 66 respondents had an additional qualification in palliative medicine/palliative care and 57 (86.4%) had at least twelve months of professional experience in palliative care. 71.2% (47 respondents) had known the SOPs prior to the survey.

### Level of implementation

#### Practical use of SOPs

The extent to which each SOP was being used actively in practice by the survey respondents ranged from a low of 22.7% (for the “Fatigue” SOP) to a highest of 48.5% (for the “Palliative Sedation” and “Respiratory Distress” SOPs). The two SOPs, which had found their way into the clinical practice of almost half of the respondents, are “Respiratory distress of an adult palliative patient “and “Palliative Sedation. With about half of the respondents (43.9%), the SOP “Acute state of confusion” was also the third most frequently used in practice.

**Fig. 1:**
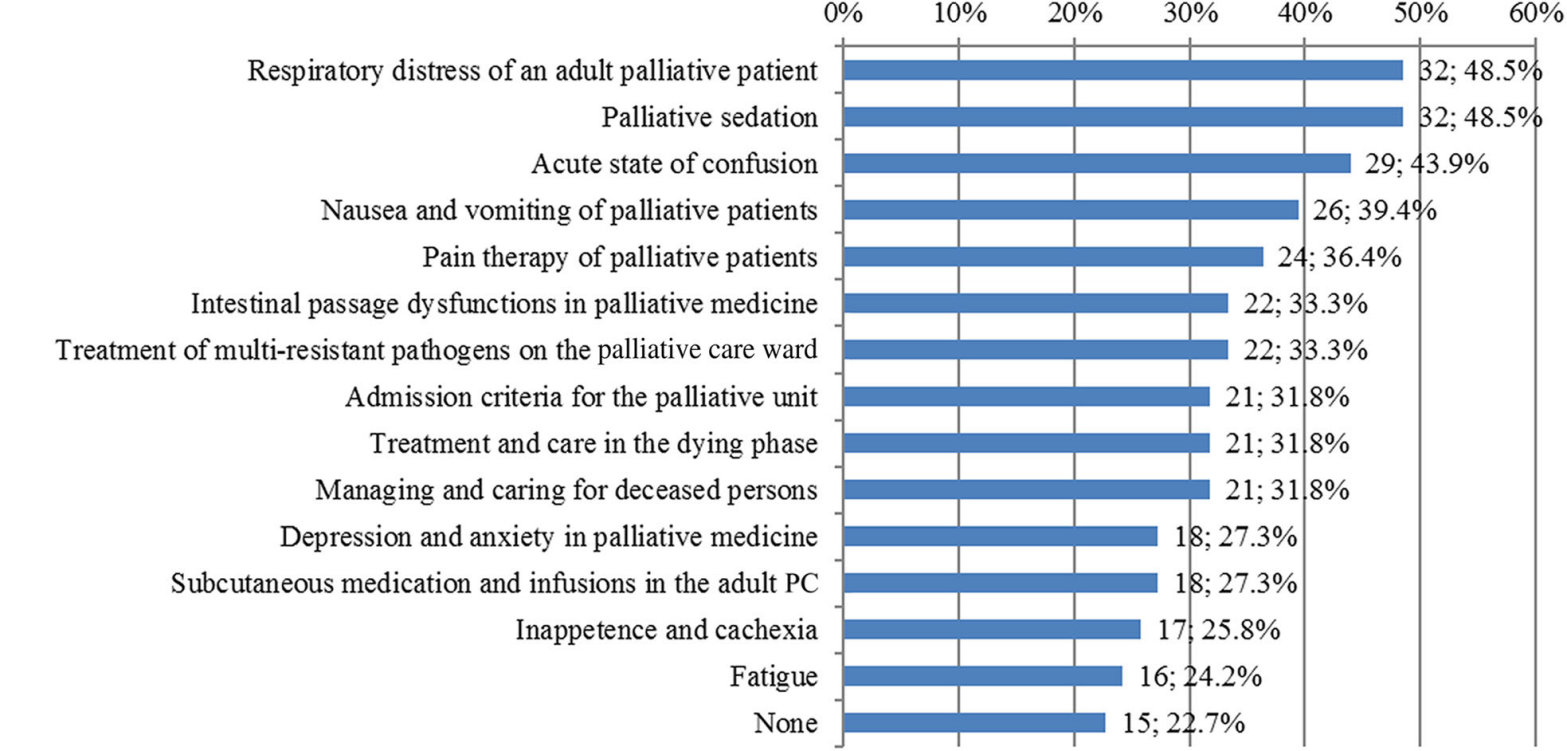
Number and percentage of respondents who are using each of the Standardized Operating Procedures in practice (*n* = 66), multiple answers possible

The lowest practical use was achieved by the SOPs “Subcutaneous medication and infusions in the adult Palliative care “ (27.3%), “Inappetence and cachexia “ (25.6%) and “Fatigue “ (24.2%). Fifteen respondents (22.7%) stated that none of the SOPs had practical use in their department, see Fig. 1.

#### SOP implementation level in the department

**Fig. 2:**
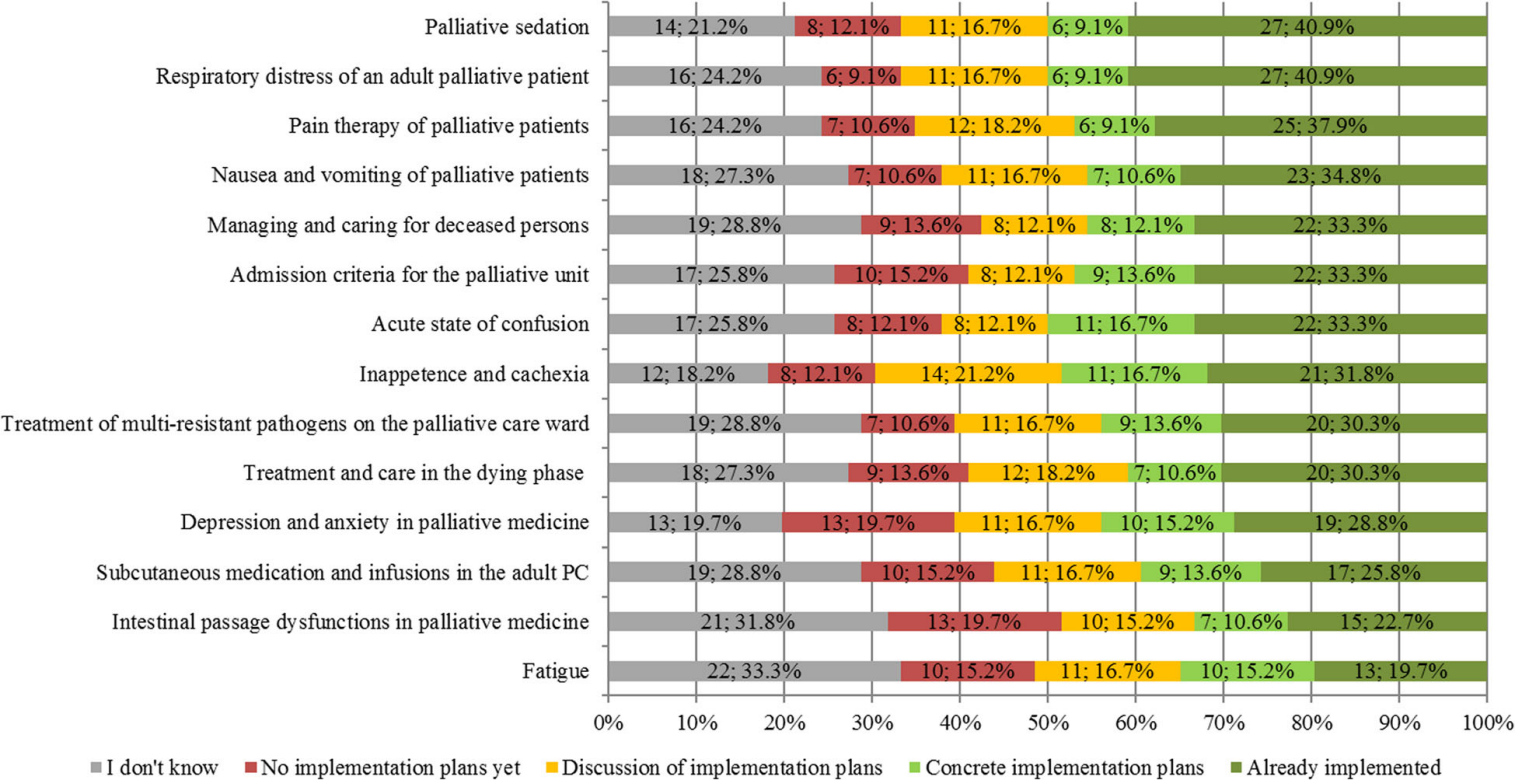
Number and percentage of current status of the implementation of the department (*n* = 66)

So far, the SOPs “Palliative sedation” and “Respiratory distress of an adult palliative patient” with 40.9%, “Pain therapy of palliative patients” with 37.9% and “Nausea and vomiting of palliative patients” with 34.8% of the respondents had already been implemented and are, therefore, the most widely used in clinical practice. Up to now, there have been no implementation plans for the SOPs “Depression and anxiety in palliative medicine”, “Intestinal passage dysfunctions in palliative medicine” with 19.7% and “Subcutaneous medication and infusions in the adult palliative medicine” as well as “Admission criteria for the palliative ward” with 15.2% each, see Fig. 2.

### Accessibility

#### Awareness of SOPs

**Fig. 3:**
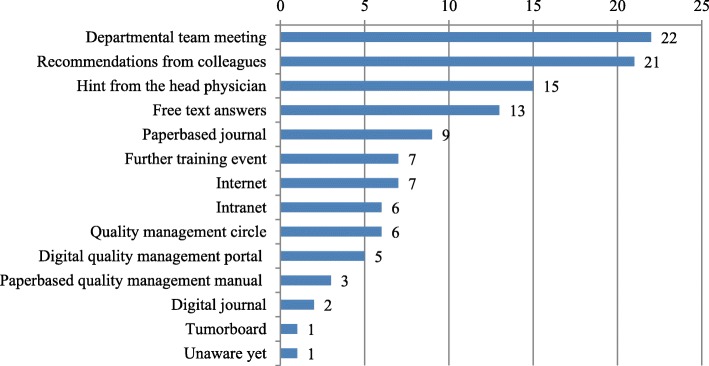
Awareness of SOPs in absolute values, multiple answers possible (*n* = 66)

Awareness of SOPs was most frequently attracted by departmental team meetings (22 responses), followed by recommendations from colleagues (21 responses) and a hint from the head physician (15 responses), see Fig. 3.

#### Knowledge of free availability

More than half (57.6%) of the respondents were aware that the SOPs for palliative care are freely available on the website of the CCC network of the German Cancer Aid.

### Frequency of use

Almost half of the respondents, 42.4% had “not yet” used the SOPs. At least 19.7% used the SOPs “quarterly”, 18.2% “weekly”, 13.6% “monthly” and 0.1% “daily”.

### Assessment of the importance, practicality and comprehensibility of SOPs

**Fig. 4:**
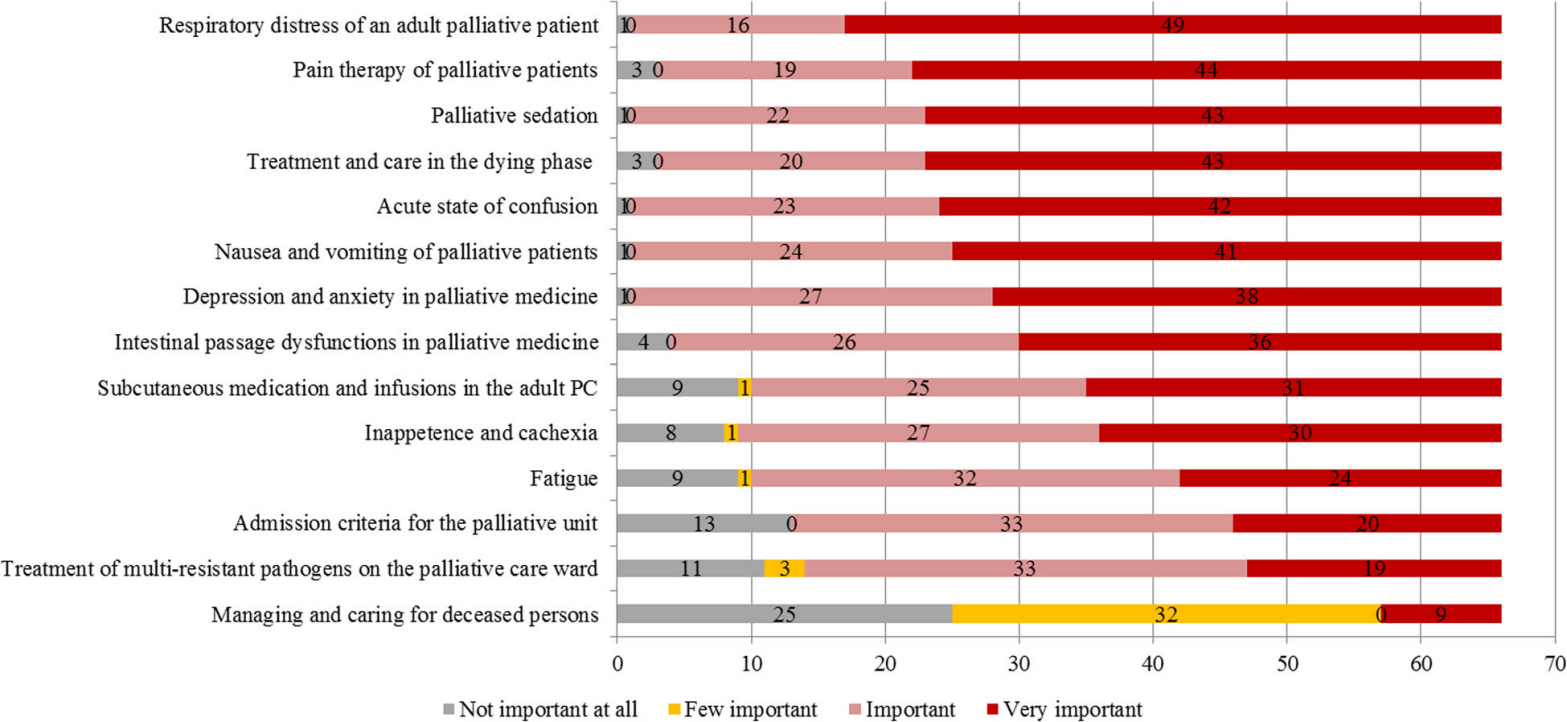
Evaluation of the importance of SOPs in absolute terms (*n* = 66)

Respondents were asked to rank each SOP as not important at all, somewhat important, important or very important. 74.2%, the majority of respondents considered the SOP “Respiratory distress in adult patients” to be very important. The SOPs “Pain therapy in palliative patients” with 66.7%, “Palliative sedation” and “Treatment and care in the dying phase” with 65.2% each were also regarded as very important. Nearly half of the respondents (48.5%) classified the SOP “Dealing with and caring for the deceased” as less important and an additional 37.9% rated it as not important at all, see Fig. 4.

Across all SOPs, about 30% of the respondents could not assess the SOPs with regard to practicality and comprehensibility, as the respondents were not aware of the SOPs. All 14 SOPs were declared understandable by almost all of the respondents; in the case of two SOPs, three respondents stated that they did not understand them.

The SOPs were rated as impractical by two to a maximum of seven of the respondents. Seven respondents declared the SOP “Treatment of multi-resistant pathogens on the palliative care ward” as impractical. Two SOPs were described by two respondents as impractical, another five SOPs by three respondents and three SOPs by four respondents. One SOP was considered impractical by five respondents, one SOP by seven and two other SOPs by six, see Table 2.

**Table 2:** Practicality

Topic	Practicality (only answered if the respondent knew the SOPs)	
	practical	impractical
Acute state of confusion	**42**	**3**
Managing and caring for deceased persons	**44**	**2**
Treatment of multi-resistant pathogens on the palliative care ward	**41**	**7**
Admission criteria for the palliative ward	**45**	**3**
Respiratory distress of an adult palliative patient	**44**	**2**
Treatment and care in the dying phase	**44**	**5**
Fatigue	**35**	**6**
Pain therapy of palliative patients	**42**	**6**
Intestinal passage dysfunctions in palliative medicine	**39**	**3**
Palliative sedation	**45**	**4**
Subcutaneous medication and infusions in the adult PC	**40**	**3**
Inappetence and cachexia	**37**	**4**
Nausea and vomiting of palliative patients	**42**	**4**
Depression and anxiety in palliative medicine	**39**	**3**

### Difficulties in SOP usage

“Finding of the SOPs” was mentioned as an inhibition by 30.3% of the respondents, and the fact that the SOPs have “no practicality in everyday life” by 12.1%. Other reasons mentioned in the free text were the lack of time or the difficulty to change common routines.

### Wishes for further SOPs

Twenty five respondents provided free text responses. The proposals for new SOPs to be developed were extremely wide-ranged. The ideas include, among others, the definition of criteria for connection to or admission to Specialized Outpatient Palliative Care, Advance Care Planning (ACP), bleeding risk, blood glucose control, stopping regular medication, a SOP which clarifies the difference between depression and fear, suicidality or handling death wishes. Four respondents indicated that the existing SOPs were sufficient.

## Discussion

In addition to the SOPs of the working group of palliative medicine within the CCC network funded by the German Cancer Aid, there are, for example, thematically similar palliative care SOPs that were developed internally at and for the Inselspital in Bern, Switzerland [23–29]. In some English-speaking countries, SOPs have been developed on how to treat dying people and on end-of-life behavior in hospice facilities [30, 31]. SOPs in palliative medicine based on the evidence- and consensus-based national S3 guideline for palliative medicine, which was developed under the leadership of the German Society for Palliative Medicine and with the first part being published in 2015 and part two updated in 2019 [1]. Similar to the evaluation of the significance of different SOPs for different symptoms, the weighting of different symptoms was also analyzed in an online study. A focus on certain symptoms such as respiratory distress and pain became apparent [32, 33]. The main topics of the SOPs may be identified not only in the S3 guideline on palliative medicine, but also in the ASCO [34] and the NCCN guidelines [35]. The implementation of the NCCN guidelines was verified and evaluated with the recommendation for appropriate measures with the aim of optimizing the level of implementation and consequently increasing the area-wide implementation [36].

So far, the SOPs in palliative medicine have not been evaluated. The authors are not aware of any national or international palliative care SOPs developed by a network of Comprehensive Cancer Centers. Therefore, the palliative care SOPs of the working group of palliative medicine and the evaluation conducted are “unique”.

A specific quality in specialized palliative medicine is the maintenance of a multi-professional team. As a result, in addition to medical and nursing staff, other professional groups are also part of the multi-professional patient care [1, 37]. The specificity of the multi-professional palliative care team allowed the SOPs to be prepared in a multi-professional and multi-disciplinary manner. The reverse conclusion can also be drawn: the use of SOPs is targeted at the entire multi-professional team.

Due to the different perspectives in view of the different professional contexts, the aim of the survey was to reflect the impression of the multi-professional team. The respondents were from all occupational groups of the multi-professional team, but the medical staff was over-represented in comparison to the other professions. The majority of respondents attained awareness of SOPs through internal sources. These included recommendations from colleagues, team meetings within the department and advice from the chief physician. External sources such as scientific journals or external events for further professional training were only mentioned by a minority. To increase awareness of and knowledge about SOPs, the sources of information need to be better used and expanded.

Although more than half of the respondents (57.6%) were previously aware of the SOPs and their availability on the website of the CCC network, awareness could be raised significantly. For example, information about the free accessibility should be provided more frequently among the teams.

By increasing the knowledge of SOPs, an even greater practical frequency of their usage is conceivable.

In addition, it is important to ensure efficient communication of the department-specific storage of the SOPs, in order to guarantee that they can be found without straining time resources. Both the intranet and the digital Quality Management portal may play a vital role here.

In assessing the importance and practical implementation, i.e. the actual application in clinical practice, individual SOPs, such as “Palliative Sedation” [15], “Acute state of confusion” [13] and “Respiratory distress of an adult palliative patient” [17] prove to be particularly significant for the participants. This is evidenced by the fact that these SOPs have already been implemented in the departments or that concrete implementation plans exist. This suggests that the need for these SOPs was greatest, either due to a lack of standardized recommendations or complex and challenging therapeutic situations.

Practicality and comprehensibility of each SOP were assessed only by those respondents having knowledge of the respective SOP. In all cases, the practicality and comprehensibility of the SOPs were assessed as positive. Consequently, there is no evidence to suggest any barriers to the practical application of these professionally prepared SOPs due to practicality and comprehensibility. Therefore, the palliative care SOPs, have the specific property of not only allowing medical staff but also nursing staff and other professional groups to understand and apply the SOPs. While many wishes for further SOPs were expressed, there was also a tone that sufficient SOPs for palliative medicine are already available in the CCC network. Accordingly, the initial focus is on updating the existing SOPs and implementing them further in practice, before expanding the existing catalogue of SOPs.

The majority of respondents stated that locating the SOPs makes them more difficult to use. Moreover, about half of the respondents used the opportunity to make their own assessments and comments regarding the difficulties of using SOPs. These focused on three main themes. One of these topics, namely the lack of information regarding the existence and location of the SOPs, confirmed the response category for difficulties locating the SOPs. A second topic is the time dimension. This includes the large amount of time required to study and understand the SOPs in addition to the already existing workload, which complicates an additional assessment of the SOPs. In this context, attention was also given to the third topic, the lack of integration of SOPs into clinical routine.

Taking these difficulties into account, the survey asked for measures that could facilitate the integration and use of SOPs in clinical practice. Additionally, an expert committee consisting of representatives from the 16 CCC locations at a meeting of the working group on palliative medicine developed a catalogue of measures aimed at increasing the awareness and use of SOPs. Emphasis is placed on internal activities such as training by colleagues or discussion in a multi-professional team as a way of getting aware of the SOPs.

This impression was also confirmed by the discussion of possible activities in the working group of palliative care in the CCC network, where weekly short training courses on SOPs integrated into existing regular team meetings were emphasized. As a result, the training on the SOPs does not require any additional time expenditure and is optimally integrated into the clinical routine. A contributing factor could also be the linking of the SOPs in connection with the request for / advise of the palliative care support team within the Hospital Information System. A pop-up window with the SOP involving the indicated symptom of the patient would be conceivable when submitting the consultation. A link of the SOPs to the website of the CCC network on the website of the corresponding palliative medicine departments seems desirable. Thereby, the SOPs may also be better introduced into generalist palliative care on the oncological wards and not only into specialized palliative care. Relevant resulting actions based on the survey and on the actions discussed above at the meeting of working group of palliative medicine, were introduced into the meeting of the working group of dying phase of the German Society for Palliative Medicine by the coordination office for Palliative Medicine in Germany. In this case, an exchange took place between medical and nursing specialists, who were present in roughly equal numbers.

Further measures could be developed as a result of the meeting of the working group for the dying phase of the German Association for Palliative Medicine in addition to the resulting measures from the survey and the discussion of the working group for palliative medicine within the CCC network.

One of these is the integration of palliative care SOPs in further education, especially in compulsory further education as well as in the teaching of students during their studies and in their final year, which is a clinical rotation. An integration of the presence of palliative care SOPs in certifications of the CCC network was also discussed.

Future research projects on palliative care SOPs should focus on optimizing awareness of SOPs not only in maximum care clinics, but also in basic and priority care clinics.

Palliative care forms of organization in the outpatient context, such as general practitioners and specialists providing outpatient palliative care, as well as outpatient hospice services and specialized outpatient palliative teams, might also benefit from the knowledge about SOPs and contribute their perspective to their development and evaluation.

### Limitations

The survey includes persons who were involved in the preparation of the SOPs and whose intention is their practical implementation. Due to data protection, it is unknown how many people of each CCC took part in the end. The survey focused to obtain an overview of all CCCs to explain the implementation status of the SOPs within the CCC network. Physicians were overrepresented in the panel, thus leaving the nursing and other professions in a minority. The aim of the working group for palliative care is to integrate these SOPs into both specialized and general palliative care. This study first started within specialized palliative care in the CCCs. The studied palliative care providers were members of specialized palliative care within the CCC network. This may have had an impact on the results. It is imaginable that less used SOPs are not used in specialized palliative care because the topic is part of their daily routine and therefore very well known. In general palliative care therefore, the topic of some SOPs may be more frequent and necessary. This needs to be further explored in the context of general palliative care and serves to further improve the SOPs. The transferability of the results is limited due to the fact that only CCCs were involved in the survey. Nevertheless, the developed measures to increase the awareness of SOPs can also be used in other settings.

## Conclusions

There is a need for measures to optimize practical implementation of the SOPs. The survey has shown that even palliative care specialists experience reasons for barriers to use such as time factors and a lack of knowledge of existence and availability. Increase of knowledge about the SOPs should be fostered through recommendations from colleagues, departmental team meetings or a hint from the head physician. Knowledge of the free availability of the SOPs on the website of the German Cancer Aid of the CCC network requires further distribution. Efforts should be made to explore facilitators for cancer care providers who are not palliative care specialists to use and adopt the SOPs. In further studies in palliative care there is a need to analyze the direct impact of SOPs on patients and the health care system in general.

## Data Availability

All data analyzed are included in this published article. Raw data set generated and analyzed during the current study is available from the corresponding author on reasonable request.

## Abbreviations

ACP: Advance care planning
CCC: Comprehensive Cancer Center
CCC network: Comprehensive Cancer Center network
CPGs: Clinical Practice Guidelines
FAU: Friedrich-Alexander-Universität Erlangen-Nürnberg
n: Sample number
SOP: Standard Operating Procedure

## References

[1] Onkologie L (2019). S3-Leitlinie Palliativmedizin für Patienten mit einer nicht-heilbaren Krebserkrankung. Lang-version 2.0, 2019, AWMF-Registernummer: 128/001OL.

[2] Stachura P, Berendt J, Stiel S, Schuler U, Ostgathe C (2017). Standard operating procedures (SOPs) in der Palliativmedizin. Schmerz.

[3] e.V. AdWMFeVA. AWMF online, Das Portal der wissenschaftlichen Medizin 2020 [Available from: https://www.awmf.org/leitlinien.html.

[4] Berendt J, Oechsle K, Thomas M, van Oorschot B, Schmitz A, Radbruch L (2016). Integration der Palliativmedizin in die von der Deutschen Krebshilfe e. V. geförderten onkologischen Spitzenzentren. DMW Deutsche Medizinische Wochenschrift.

[5] Ostgathe C, Thomas M, Berendt J (2017). Standard operating procedures (SOPs) for palliative care of patients in the network of German Comprehensive Cancer Centers (CCCs).

[6] Ahrens M, Hornemann B, Viehrig M, Berendt J, Gog C (2017). SOP–Umgang mit und Versorgung von Verstorbenen. Onkologe.

[7] Cuhls H, Mücke M, Jaspers B, Jentschke E, Hense J, Wolf C (2017). SOP–fatigue. Onkologe.

[8] Eschbach C, Stachura P, Villalobos M, Wolf C, Thomas M (2017). SOP–Inappetenz und KachexieSOP–inappetence and cachexia. Onkologe.

[9] Ettrich T, Schönsteiner S, Mayer-Steinacker R, Döhner H, Gog C, Thuss-Patience P (2017). SOP–Darmpassagestörung in der PalliativmedizinSOP—bowel dysfunction in palliative care medicine. Onkologe.

[10] Ettrich T, Schönsteiner S, Mayer-Steinacker R, Döhner H, Gog C, Thuss-Patience P (2017). Erratum zu: SOP–Darmpassagestörung in der PalliativmedizinErratum to: SOP–bowel dysfunction in palliative care medicine. Onkologe.

[11] Gärtner J, Jaroslawski K, Thuss-Patience P, Rosenbruch J, Berendt J, Becker G (2017). SOP–Aufnahmekriterien auf die Palliativstation. Onkologe.

[12] Hense J, Przyborek M, Rosenbruch J, Ostgathe C, Wolf C, Bogner S (2017). SOP–Subkutane Medikamentengabe und Infusionen in der erwachsenen PalliativmedizinSOP—subcutaneous drug administration and infusions in adult palliative medicine. Onkologe.

[13] Jentschke E, Thomas M, Babiak A, Lewerenz J, Oechsle K, van Oorschot B (2017). SOP–Akuter Verwirrtheitszustand. Onkologe.

[14] Montag T, Starbatty B, Thomas M, Gog C, Ostgathe C, Simon S (2017). SOP–Behandlung und Betreuung in der Sterbephase. Onkologe.

[15] Oechsle K, Radbruch L, Wolf C, Ostgathe C (2017). SOP–palliative SedierungSOP—palliative sedation. Onkologe.

[16] Ostgathe C, Stachura P, Hofmann S, van Oorschot B, Oechsle K, Bogdan C (2017). SOP–Umgang mit multiresistenten Erregern auf der Palliativstation. Onkologe.

[17] Rosenbruch J, Eschbach C, Viehrig M, Ostgathe C, Bausewein C (2017). SOP–Atemnot bei erwachsenen Palliativpatienten. Onkologe.

[18] Schwartz J, Neukirchen M, De Vilder M-C, Hornemann B, Wolf C, Gärtner J (2017). SOP–depression und angst in der PalliativmedizinSOP—depression and anxiety in palliative medicine. Onkologe.

[19] Thuss-Patience P, Markwordt J, Mayer-Steinacker R, Ettrich T, Wolf C, Stachura P (2017). SOP–Übelkeit und Erbrechen bei PalliativpatientenSOP–nausea and vomiting in palliative patients. Onkologe.

[20] Viehrig M, Schlisio B, Thomas M, Gärtner J, Wolf C, Hense J (2017). SOP–Schmerztherapie bei PalliativpatientenSOP—pain therapy in palliative care patients. Onkologe.

[21] Ostgathe C, Thomas M (2018). SOP - Palliativmedizin. Onkologe.

[22] Krebshilfe SD. Palliative Netzwerk-SOPs 2019 [Available from: http://www.ccc-netzwerk.de/netzwerk-sops/palliative-netzwerk-sops.html.

[23] Eychmüller S, Fliedner M (2016). Schmerztherapie Specials in Palliative Care, Standard Operating Procedure (SOP).

[24] Eychmüller S, Fliedner M (2016). Nausea und Erbrechen, Standard Operating Procedure (SOP).

[25] Eychmüller S, Fliedner M (2016). Fatigue-Müdigkeit in der Palliative Care, Standard Operating Procedure (SOP).

[26] Eychmüller S, Fliedner M (2016). Delir (synonym: akuter Verwirrtheitszustand), standard operating procedure (SOP).

[27] Eychmüller S, Fliedner M (2016). Atemnot und terminale Rasselatmung, Standard Operating Procedure (SOP).

[28] Eychmüller S, Mösli P (2016). Kulturell-religiöse Besonderheiten am Lebensende, Standard Operating Procedure (SOP).

[29] Fliedner M (2016). DOLS pc. Obstipation, Standard Operating Procedure (SOP).

[30] Consultant in Palliative Care / Head of Division ES. END OF LIFE CARE POLICY. Somerset Partnership End of Life Best Practice Group; 2017.

[31] Young C. Care of dying adults in the last days of life. Improving care at the very end of life: Hospice Isle Of Man: Department of Health and Social Care/Isle of Man Government; 2018.

[32] Kalies H, Schottmer R, Simon ST, Voltz R, Crispin A, Bausewein C (2017). Critical attitudes and beliefs towards guidelines amongst palliative care professionals - results from a national survey. BMC Palliat Care.

[33] Kalies H, Schottmer R, Simon ST, Voltz R, Crispin A, Bausewein C (2018). Barriers for the implementation of guidelines in palliative care-results from a national survey of professionals. Supp Care Cancer.

[34] Osman H, Shrestha S, Temin S, Ali ZV, Corvera RA, Ddungu HD (2018). Palliative care in the global setting: ASCO resource-stratified practice guideline. J Global Oncol.

[35] Dans M, Smith T, Back A, Baker JN, Bauman JR, Beck AC (2017). NCCN guidelines insights: palliative care, version 2.2017. J Natl Compr Canc Netw.

[36] Albizu-Rivera A, Portman DG, Thirlwell S, Codada SN, Donovan KA (2016). Implementation of NCCN palliative care guidelines by member institutions. Supp Care Cancer.

[37] CCC-Netzwerk APi. Best Practice- Empfehlungen zur Integration der Palliativmedizin in ein von der Deutschen Krebshilfe gefördertes Comprehensive Cancer Center (CCC). URL: http://www.ccc-netzwerkde/arbeitsgruppen/palliativmedizin.html. 2017.

